# Supplementary figures and images for: UK Medical Cannabis Registry: A clinical outcomes analysis for insomnia
Source: PLOS Ment Health. 2025 Aug 27;2(8):e0000390. doi: 10.1371/journal.pmen.0000390 (PMC12798294; doi:10.1371/journal.pmen.0000390)

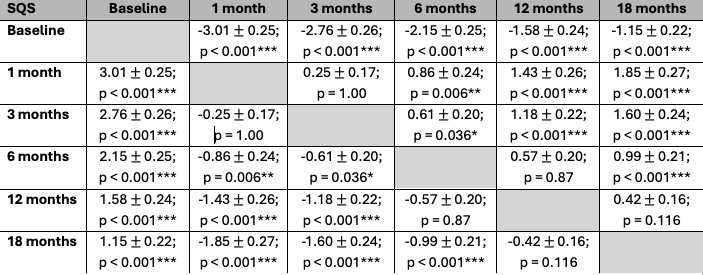
**S1 Appendix:**

Supplement: S1 Appendix — The values represent the mean difference ± standard error. *p < 0.050, **p < 0.010, ***p < 0.001. d - Cohen’s d. (DOCX) [file pmen.0000390.s001.docx]

**S2 Appendix:**


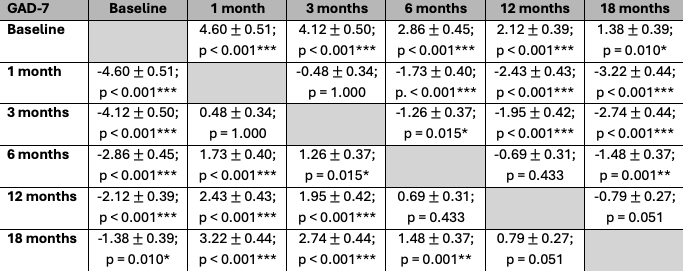

Supplement: S2 Appendix — The values represent the mean difference ± standard error. *p < 0.050, **p < 0.010, ***p < 0.001. d - Cohen’s d. (DOCX) [file pmen.0000390.s002.docx]

**S3 Appendix:**


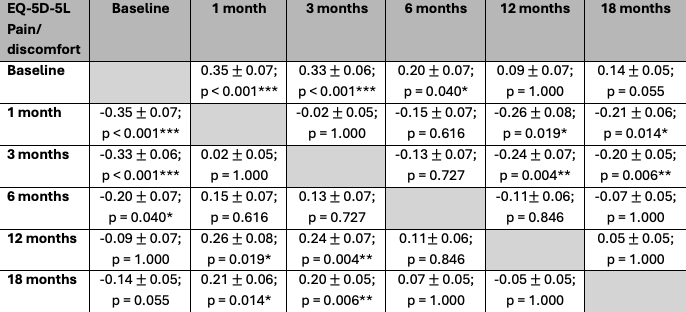

Supplement: S3 Appendix — The values represent the mean difference ± standard error. *p < 0.050, **p < 0.010, ***p < 0.001. d - Cohen’s d. (DOCX) [file pmen.0000390.s003.docx]

**S4 Appendix:**


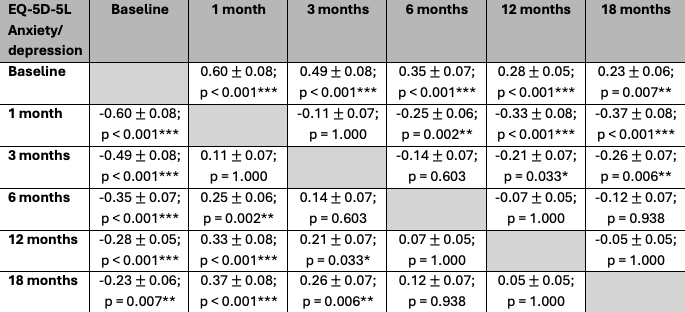

Supplement: S4 Appendix — The values represent the mean difference ± standard error. *p < 0.050, **p < 0.010, ***p < 0.001. d - Cohen’s d. (DOCX) [file pmen.0000390.s004.docx]

**S5 Appendix:**


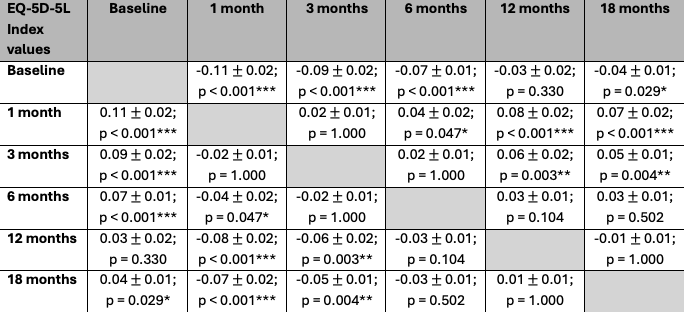

Supplement: S5 Appendix — The values represent the mean difference ± standard error. *p < 0.050, **p < 0.010, ***p < 0.001. d - Cohen’s d. (DOCX) [file pmen.0000390.s005.docx]

**S6 Appendix:**


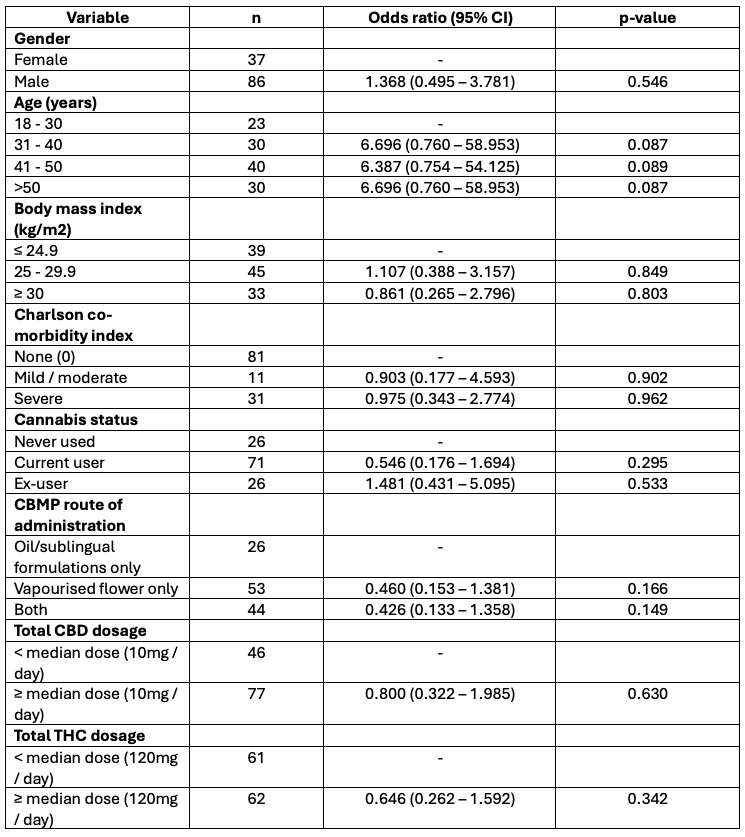

Supplement: S6 Appendix — A univariate logistic regression model was utilised to conduct statistical analysis. n = 123. CBD – cannabidiol; CBMP – cannabis-based medicinal product; THC - Δ9-tetrahydrocannabinol. (DOCX) [file pmen.0000390.s006.docx]

**S7 Appendix:**


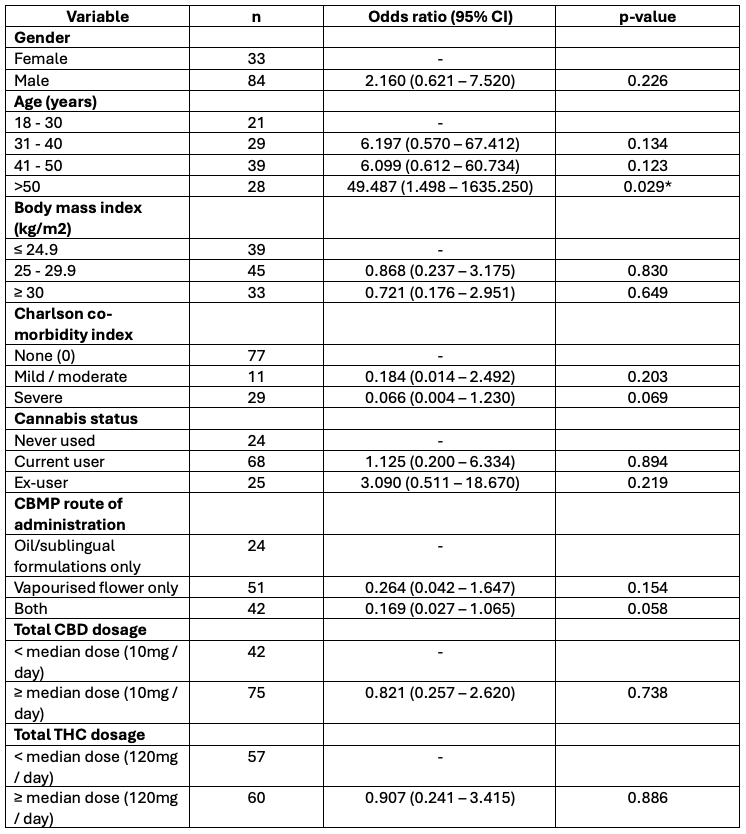

Supplement: S7 Appendix — A multivariate logistic regression model was utilised to conduct statistical analysis. *p < 0.050. n = 117. CBD – cannabidiol; CBMP – cannabis-based medicinal product; THC - Δ9-tetrahydrocannabinol. (DOCX) [file pmen.0000390.s007.docx]

**S8 Appendix:**


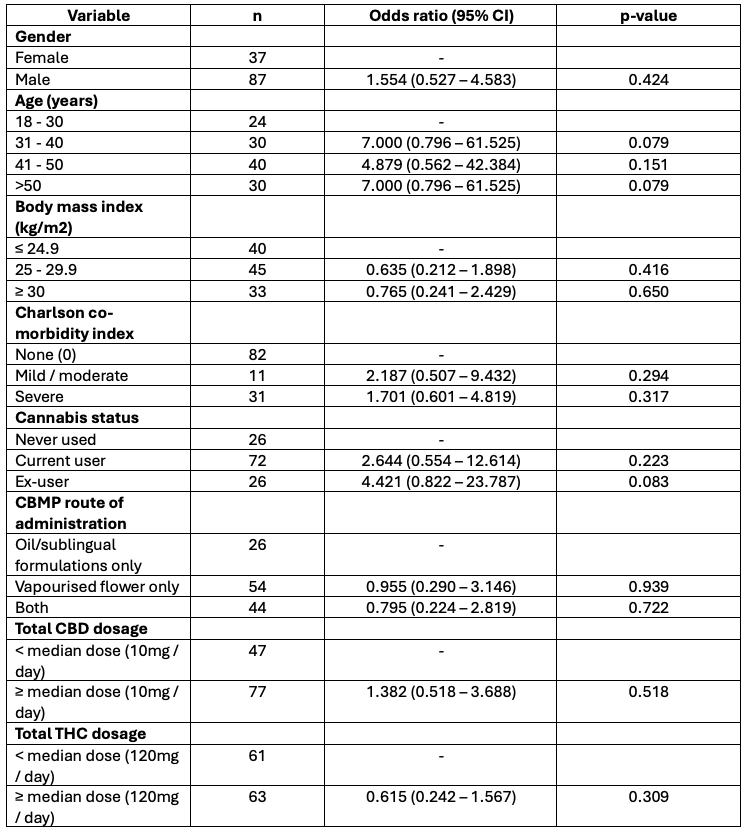

Supplement: S8 Appendix — A univariate logistic regression model was utilised to conduct statistical analysis. n = 124. CBD – cannabidiol; CBMP – cannabis-based medicinal product; THC - Δ9-tetrahydrocannabinol. (DOCX) [file pmen.0000390.s008.docx]

**S9 Appendix:**


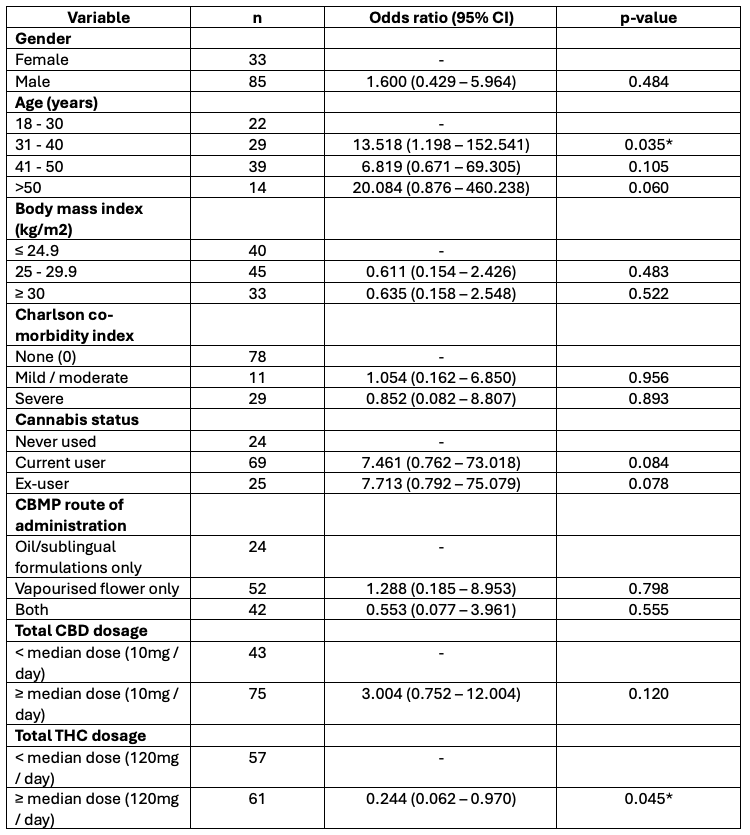

Supplement: S9 Appendix — A multivariate logistic regression model was utilised to conduct statistical analysis. *p < 0.050. n = 118. CBD – cannabidiol; CBMP – cannabis-based medicinal product; THC - Δ9-tetrahydrocannabinol. (DOCX) [file pmen.0000390.s009.docx]
