# Supplementary material for: UK Medical Cannabis Registry: A clinical outcomes analysis for insomnia
Source: PLOS Ment Health. 2025 Aug 27;2(8):e0000390. doi: 10.1371/journal.pmen.0000390 (PMC12798294; doi:10.1371/journal.pmen.0000390)
Supplement: Attachment — Submitted filename: Response to Reviewers.docx [file pmen.0000390.s011.docx]

*21^st^ April 2025*

**Dear Editors and Reviewers,**

Thank you very much for your extensive review and the kind opportunity to resubmit our project for consideration in your distinguished journal. Please see below for a line-by-line response to each comment and indications where we have made changes in the manuscript.

**Referee 1**
**Comment 1:** To say that there is no data supporting the long term efficacy and safety of Z-drugs is incorrect. Please cite the 6-month RCTs on eszopiclone.

**Response 1 :** Thank you for your thoughtful comment. We have revised the manuscript to acknowledge the existence of the 6-month RCTs on eszopiclone.

*“While the short-term efficacy of benzodiazepines and Z-drugs is well supported, evidence for long-term benefit is limited. Six-month RCTs of eszopiclone offer some support^32^, but concerns about dependence, withdrawal symptoms and other adverse effects remain^33-36^.*

**Comment 2:** The dual orexin receptor antagonists were completely overlooked as a treatment option and should be described as a safer alternative to z drugs and maybe safer than any form of cannabis.

**Response 2:** We thank the reviewer for highlighting this important omission. We have now included a brief discussion of dual orexin receptor antagonists, and noted their favourable safety profile compared to Z-drugs, as well as their current limitations:

*“More recently, dual orexin receptor antagonists have been introduced as a novel class of hypnotics. These agents promote sleep by inhibiting wakefulness, and early evidence suggests they may offer a favourable safety profile compared to traditional GABAergic medications^43,44^. However, many of these medications are unavailable in Europe. Daridorexant, the only orexin antagonist available in Europe, has recently been approved by the National Institute for Health and Care Excellence as a second-line treatment for insomnia disorder^45^. However, the use of this medication class remains limited by cost, availability, and safety data^46,47^.”*

**Comment 3:** There is a mention of a review of CBD/THC in 34 included studies, showing "benefit in a subset of patients". Please clarify the characteristic of the subset which benefited and who did not benefit

**Response 3:** We thank the reviewer for this helpful comment. To clarify, the systematic review of 34 studies did not identify a specific subgroup of patients who benefited from treatment. Rather, it reported that each study documented improvements in insomnia symptoms in some participants. However, the characteristics of these participants were not consistently described across studies, making it difficult to draw conclusions about which individuals are most likely to benefit. We have revised the manuscript to reflect this more accurately.

*“A systematic review of 34 studies found that every study documented improvements sleep quality among a portion of their participants^62^. This review considered patients receiving treatment for conditions other than insomnia and there was no subgroup analysis, again prohibiting identification of the optimal cannabis-based medicinal products for treatment of insomnia.”*

**Comment 4:** The authors correctly describe the initial improvement then followed by a noticeable regression of benefit in the SQS and GAD7 scores, suggesting that this might represent tolerance. This impression is further underscored by the fact that CBD and THC doses are advancing at a time that SQS and GAD7 scores are regressing. The authors should explicitly link these trends as further evidence of possible tolerance of effect.

**Response 4:** We thank the reviewer for this valuable observation. We agree that the temporal association between increasing CBD and THC doses and the reduction in treatment effect provides further evidence for possible pharmacological tolerance. We have now explicitly linked these trends in the manuscript, noting that the increase in dosage over time may reflect dose escalation in response to diminishing therapeutic effects, consistent with the development of tolerance:

*“Notably, this apparent reduction in treatment effect over time coincided with increasing doses of both CBD and THC, suggesting that participants may have developed tolerance to the therapeutic effects of cannabis-based medicinal products. This dose-response shift further supports the possibility of pharmacological tolerance contributing to the diminishing improvements observed in SQS and GAD-7 scores.*

**Referee 2**

**Comment 1:** Please note that p = 0.05 is not significant per the traditional threshold. Correct this throughout and update abstract.

**Response 1 :** Thank you for your comment. There may be some confusion as we have stated that p<0.050 is the threshold presented in the study for statistical significance, in accordance with traditional thresholds.

*“Statistical analyses were conducted using the Statistical Package for the Social Sciences (SPSS; v.29.0.0.0), while GraphPad Prism (v. 9.4.1(350)) was utilised for graph creation. P<0.050 indicated statistical significance.”*

**Comment 2:** The acronym CBMP is not commonly used and detracts from the work. Please reconsider using this unfamiliar acronym.

**Response 2:** Thank you very much for this insight and apologies. It is a commonly used abbreviation in the UK amongst clinical guidance, but appreciate this may not be well known by all. Instead, we have dropped use of the acronym altoghether to try and improve readability.

**Comment 3:** The authors cite that a systematic review of 34 studies found a uniform improvement in insomnia symptoms “in a sub-set of participants”. This is a bit misleading as only 2 of the 34 studies focused on patients with insomnia, and others were just poor sleep etc. Please revise this to more accurately reflect the results of the review and the samples used.

**Response 3 :** Thank you for your comment. We apologise that this was unintentionally misleading. We have made some alterations to the study as follows to reflect the suggested changes:

*“A systematic review of 34 studies found that every study documented improvements sleep quality among a portion of their participants^62^. This review considered patients receiving treatment for conditions other than insomnia and there was no subgroup analysis, again prohibiting identification of the optimal cannabis-based medicinal products for treatment of insomnia.”*

**Comment 4:** While I understand why no subgroup analyses were performed on patient characteristics (e.g. anxiety), what about cannabis use characteristics (e.g. administration route, THC dose etc.). Authors state that this information was available but appears to be missing from analyses

**Response 4 :** Thank you very much for your comment. We did include administration route and CBD/THC dose in our univariate and multivariate analysis, alongside gender, age, BMI and Charlson comorbidity index. This is detailed in our section on univariate and multivariate analysis in the results. Due to limitations of word count and table/figure counts we haven’t been able to report all outcomes in the main manuscript, but Appendix F, G, H, and I contain additional information about these outcomes.

**Comment 5:** Was information available on the sub-type of insomnia diagnosed? If not, can the authors speculate on how this might affect results and provide further discussion of this.

**Response 5 :** Thank you very much for your comment. Unfortunately this information was not available, so we have expanded on this in the limitations section of the discussion.

*“A systematic review of 34 studies found that every study documented improvements sleep quality among a portion of their participants^62^. This review considered patients receiving treatment for conditions other than insomnia and there was no subgroup analysis, again prohibiting identification of the optimal cannabis-based medicinal products for treatment of insomnia.”*

**Comment 6 - 10:** I recommend that the authors reconduct their analyses using a more sophisticated Linear Mixed Effect Model with appropriate covariance structure, stratified by administration route. This will provide more sensitive tool to assess both time and participant-level factors for the cannabis treatments, accounting for repeated assessment timepoints.

I am surprised that analyses did not control for baseline characteristics, such as cannabis dose and/or administration. Please include these when performing re-analysis.

Can the authors provide more justification for not stratifying the results by treatment modality, such as administration route? Given that previous work has shown at least some effect differences for those who consume an oil-based product vs dried flower for sleep benefits, and as this information was seemingly available to the authors, it is surprising that this was not considered.

Provide a measure of effect size for all the reported analyses (and for any updated analyses that are performed in subsequent revisions

**Response 6-10 :** Thank you very much for your comments on our methodology and analysis. We have aggregated these comments for one response as they all relate to the same topic.

In replace of the repeated measures ANOVA we have followed your kind suggestion to use a linear mixed effects model. Consequently we have updated all results, tables and discussion with this updated information. The most meaningful change is that the EQ-5D-5L Usual activities was no longer statistically significant. Therefore post-hoc pairwise comparisons were removed. There were some changes to recorded p-values in other PROMs, but all other values remained statistically significant.

We have also added in the F statistic, degrees of freedom and effect size (η^2^ / cohen’s d) for our analyses.

In relation to stratification, because patients may move between different medications during the course of treatment we preferred to deal with this by assessing the relationship between treatment a patient was receiving at 18 months and the likelihood of reporting a minimal clinically significant difference in the validated SQS and GAD-7 scales. In our multivariable analysis we account for gender, age, BMI, Charlson comorbidity index, prior cannabis use, route of administration, total CBD dose at 18 months, and THC dose at 18 months.

I hope you can see our rationale for keeping this particular stratification the same in the resubmission.

*“To assess changes in PROM scores across the analysis, a linear mixed effects model was utilised. Effect size was reported as η^2^ values. Subsequently, significant findings linear mixed effects model underwent post-hoc pairwise comparisons, with the application of Bonferroni correction. Effect size for pairwise comparisons were reported as cohen’s d values and interpreted as small (d ≥ 0.2), medium (d ≥ 0.5), and large (d ≥ 0.8), respectively^102^. This analysis diminished the likelihood of type I errors^103,104^. PROM data was considered parametric, based on the central limit theorem^105^.”*

*“A linear mixed effects model was utilised to compare PROM scores across all recorded time periods* ***(Table 5)****. Single-Item Sleep Quality Score (SQS) improved from baseline (2.66 ± 2.41) to 1- (5.67 ± 2.65; p<0.001; d=1.07), 3- (5.41 ± 2.69; p<0.001; d=0.96), 6- (4.80 ± 2.89; p<0.001; 0.76), 12- (4.24 ± 3.01; p<0.001; d=0.59) and 18- (3.81 ± 2.90; p<0.001; d=0.47) months* ***(Figure 2a; Appendix A)****. Generalised Anxiety Diorder-7 (GAD-7) scores improved from baseline (9.59 ± 6.31) to 1-month (4.99 ± 4.91; p<0.001; d=0.81). The differences between baseline and follow-up months 3, 6, 12 and 18 were less pronounced over time, but still statistically significant (p<0.050)* ***(Figure 2b; Appendix B)****. The EuroQol-5 Dimension-5 Level (EQ-5D-5L) dimensions of pain/discomfort (p=0.036), anxiety/depression (p=0.001), and index values (p<0.001) all demonstrated significance. All pairwise comparisons for these PROMs are detailed in full in* ***Appendix C – E****. However, no significant changes were observed in the EQ-5D-5L mobility (p = 0.966), self-care (p = 0.983), or usual activities (p=0.208) dimensions. Patient Global Impression of Change (PGIC) scores did not show significant differences across the follow-up period (p = 0.591).”*

*Also we kindly draw your attention towards Table 5 in the main manuscript and Appendix A-E which have all been ammended.*

**Comment 11:** Was any data collected on recreational cannabis usage?

**Response 11:** Many thanks for your comment. Data was collected on prior cannabis consumption, but ongoing non-prescribed cannabis consumption was not recorded as patients were counselled to stop all other sources of cannabis whilst prescribed cannabis-based medicinal products. We have tried to make this clearer, as follows:

*“Data on alcohol consumption and tobacco use was collected. Cannabis status was categorised as: ‘current user,’ ‘ex-user,’ or ‘never used.’ To measure lifetime cannabis consumption among current and ex-users, the gram years metric was used. This metric, utilised in previous UKMCR studies^86-88^, is determined by multiplying the average daily cannabis consumption in grams by the number of years of use^89^. Patients were counselled to stop external sources of cannabis during treatment with CBMPs, hence cannabis consumption outside of legally prescribed use was not recorded.”*

Kind Regards,

Study Authors
