# Supplementary material for: UK Medical Cannabis Registry: A clinical outcomes analysis for insomnia
Source: PLOS Ment Health. 2025 Aug 27;2(8):e0000390. doi: 10.1371/journal.pmen.0000390 (PMC12798294; doi:10.1371/journal.pmen.0000390)
Supplement: Attachment — Submitted filename: Response to Reviewers 2.docx [file pmen.0000390.s012.docx]

*12^th^ June 2025*

**Dear Editors and Reviewers,**

Thank you very much for your extensive review and the kind opportunity to resubmit our project for consideration in your distinguished journal. Please see below for a line-by-line response to each comment and indications where we have made changes in the manuscript.

**Referee 2**
**Comment 1:** Thank you for your careful review of the statistical approach. The application of LMM has strengthened the work, however key details from this approach are still missing. Can the authors please provide:

i.) how model selection was guided

ii.) what covariance structure was used and why

iii.) the random factor

iv.) the fixed factor(s)

v.) whether separate models were build per outcome

vi). whether both main effect and interactions were explored, and in what order

**Response 1 :** Many thanks for your comment. We have now included further information on this in the methods section as follows:

*‘To assess changes in PROM scores across the analysis timepoints, separate linear mixed effects models were constructed for each outcome measure. Model selection was guided by the longitudinal nature of the data and the need to account for within-subject correlation.*

*The models employed a random intercept structure with compound symmetry covariance, which assumes equal correlations between all timepoints within subjects. This covariance structure was selected as appropriate for the repeated measures design where measurements were taken at regular intervals. The random factor was the unique participant identifier allowing for individual baseline differences between participants. Time was included as the primary fixed factor.*

*Separate models were constructed for each PROM outcome to account for the distinct measurement properties and clinical interpretations of each scale. Initially, main effects models were fitted to examine the overall effect of time on each outcome. Given the primary research question focused on temporal changes, interaction terms were not explored in this analysis. Effect size was reported as η2 values. Subsequently, significant findings linear mixed effects model underwent post-hoc pairwise comparisons, with the application of Bonferroni correction. Effect size for pairwise comparisons were reported as Cohen’s d values and interpreted as small (d ≥ 0.2), medium (d ≥ 0.5), and large (d ≥ 0.8), respectively^102^. This analysis diminished the likelihood of type I errors^103,104^. PROM data was considered parametric, based on the central limit theorem^105^.’*

Kind Regards,

Study Authors
